# Tea Harvesting and Processing Techniques and Its Effect on Phytochemical Profile and Final Quality of Black Tea: A Review

**DOI:** 10.3390/foods12244467

**Published:** 2023-12-13

**Authors:** Muhammad Aaqil, Chunxiu Peng, Ayesha Kamal, Taufiq Nawaz, Fei Zhang, Jiashun Gong

**Affiliations:** 1 College of Food Science and Technology, Yunnan Agricultural University, Kunming 650201, China; aaqilkhan26@gmail.com (M.A.); zhangfei92022@163.com (F.Z.); 2 College of Horticulture and Landscape, Yunnan Agricultural University, Kunming 650201, China; pengchunxiu2023@163.com (C.P.); ayeshakamal235@gmail.com (A.K.); 3 College of Natural Sciences, South Dakota State University, Brookings, SD 57007, USA; taufiq.nawaz@jacks.sdstate.edu; 4 Agro-Products Processing Research Institute, Yunnan Academy of Agricultural Sciences, Kunming 650221, China

**Keywords:** black tea, processing technique, phytochemical profile, sensory attributes

## Abstract

Tea (*Camellia sinensis*) has grown for over 300 years and is recognized worldwide as among other well-renowned crops. The quality of black tea depends on plucking (method, standard, season, and intervals), withering and rolling (time and temperature), fermentation (time, temperature, and RH), drying (temperature and method), and storage conditions, which have a high influence on the final quality of black tea. At the rolling stage, the oxidation process is initiated and ends at the early drying stage until the enzymes that transform tea polyphenols into thearubigins (TRs) and theaflavins (TFs) are denatured by heat. By increasing fermentation time, TRs increased, and TF decreased. Each is liable for black tea’s brightness, taste, and color. The amino acids and essential oils also grant a distinctive taste and aroma to black tea. Throughout withering, rolling, and fermentation, increases were found in essential oil content, but during drying, a decrease was observed. However, the Maillard reaction, which occurs when amino acids react with sugar during drying, reimburses for this decrease and enhances the flavor and color of black tea. As compared to normal conditions, accelerated storage showed a slight decrease in the total color, TF, and TRs. It is concluded that including plucking, each processing step (adopted technique) and storage system has a remarkable impact on black tea’s final quality. To maintain the quality, an advanced mechanism is needed to optimize such factors to produce high-quality black tea, and an objective setting technique should be devised to attain the desirable quality characteristics.

## 1. Introduction

Tea, scientifically known as (*Camellia sinensis* L.), is a widely consumed beverage worldwide due to its satisfactory aroma, taste, and health benefits [1,2]. Tea consumption originated in ancient China, where it was served as a beverage and medicine [3]. Approximately 75% of tea consumption in the world comprises black tea, which accounts for the highest production and consumption [4]. The ambient temperature range required for a tea plant is 18 °C to 25 °C to grow well. Shoot growth is observed to be slowed down if the temperature is under 13 °C or above 30 °C. Tea plants require at least 1200 mm of rainfall annually. The optimal soil pH range for tea plant growth is 4.5–5.6. The best soil condition for the growth of tea plants is deep, having greater than 2% organic matter, well aerated and drained [5]. Usually, it is made from young shoots with one unopened bud and two or three leaves. Tea has been a very trendy beverage for recreation and pleasure for centuries and is also regarded as a nutritious beverage due to its therapeutic and antioxidant properties [6]. Predominantly, the tea plant originated in Southeast Asian countries and has since been cultivated in more than thirty countries around the world, encompassing both regions, including tropical and subtropical [7,8]. The total annual production and consumption of tea is three billion kilograms in the world [9].

The production of black tea is carried out by tea leaves experiencing several physicochemical reactions during different processing steps [10]. There are various kinds of teas in the market, such as yellow, red, green, black, and so on with a distinct fermentation, oxidation, and processing method [11]. As a fermented tea, black tea is typically produced in two ways orthodox and crush, tear, and curl (CTC). The orthodox involves four stages: withering, maceration or rolling, fermentation, and drying, whereas CTC black tea processing involves withering, maceration, cutting, tearing and curling, fermentation, and drying. The CTC process has a horizontal or vertical Rotorvane maceration machine for the maceration of tea leaves instead of rollers [12]. The most important of these stages is fermentation, which affects the smell, color, mouth feel, and, most importantly, the bio functions of black tea [13,14].

**Figure 1:**
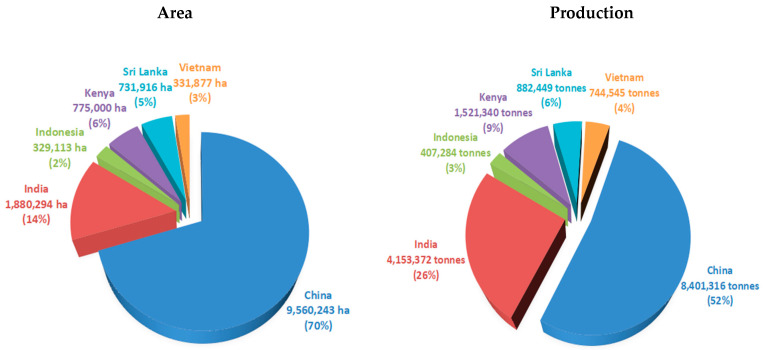
Tea plantation area and production among six countries during the years 2018, 2019, and 2020.

Around the world, the leading countries that produce tea are in the Asian continent [15]. China, India, Kenya, Indonesia, Sri Lanka, and Vietnam are the primary producers, accounting for around 77% of the global tea production and 80% of exports [16]. According to the database of the Food and Agriculture Organization (FAO) of the United Nations [17], China was the leading tea-planting and tea-producing country among India, Sri Lanka, Indonesia, Vietnam, and Kenya in 2018, 2019, and 2020, which accounted for around 70% and 52% of plantation area and production, respectively (Figure 1). Tea is primarily cultivated in tropical countries in Africa, with Kenya being the leader in tea production and export. Over 10% of the population in Kenya relies on it as a source of income [18]. It is also the country’s primary source of income, with more than 10 million people relying on it directly or indirectly [19].

Black tea’s quality is widely determined by the physical and chemical procedures used in its production since tea leaves undergo many chemical transformations. Therefore, depending on the technique followed during the processing of black tea, the phytochemical profile may vary significantly. Tea quality is primarily influenced by leaf variety (*Camellia sinensis* L./*Camellia sinensis assamica*), growth conditions, plucking protocol, interval and season, manufacturing processes, infusion preparation, and ground tea leaf size. Leaf appearance briskness, aroma, color, flavor, and liquor brightness are considered for quality determination [20,21,22]. Tea’s primary quality features are color, taste, and aroma [23]. Consumers highly prefer nutritional food. Tea processing needs to be performed under strict supervision to preserve its desired qualities, including its natural taste, appearance, aroma, color, and health benefits.

Numerous reviews have emphasized the different processing steps, chemical composition, functional properties, and health benefits of black tea. As per our information, no review has been carried out in relation to the existing evidence on the handling (plucking method, standard, interval, and season) and processing techniques adopted during the manufacturing of tea and their influence on its phytochemical and sensory profile. The main objective of our study is to examine how handling and processing affect the phytochemical and sensory qualities and the key components linked with the phytochemical attributes of black tea. Understanding and regulating the various stages of tea processing and handling are crucial; therefore, this study aims to shine the spotlight on existing literature and research about the various scientific and technological factors involved in the processing and handling of black tea in order to adopt the most suitable handling and processing techniques in future to produce high-quality black tea.

## 2. Black Tea’s Health Benefits and Chemical Composition

Tea is abundant in amino acids, caffeine, polyphenols, and other functional elements that have been connected to a variety of health benefits, including anti-inflammation, antioxidation, anti-mutagenic, anti-cancer, and enhancement of psychomotor performance [24,25,26,27]. Any changes in the aforementioned composition of phytochemicals affect the quality of black tea [28]. To determine tea consumption’s potential in avoiding different oxidative-stress-related chronic diseases like diabetes, Alzheimer’s disease, cardiovascular conditions, and some cancers, numerous in vitro, in vivo human, and animal intervention studies have been conducted [29,30]. Tea phytochemicals, notably polyphenols, are primarily responsible for these protective effects [31]. Previous research has demonstrated that black tea’s flavonoid consumption favorably affects coronary circulation [32] and can reduce endothelial dysfunction [33], even though individual differences in flavonoid metabolism may influence the latter [34]. Flavonoids of tea were additionally demonstrated to decrease low-density lipoprotein (LDL) cholesterol levels by 11.1% [35]. Animal and in vitro research exhibited effects beyond antioxidant capacity, such as decreased endothelial adhesion molecule expression [36]. Before the British turned tea into a drink in the 19th century, India utilized it as a medicine [37]. That is why research interest in tea has experienced a notable increase in recent years due to its potential health advantages for humans [38].

**Figure 2:**
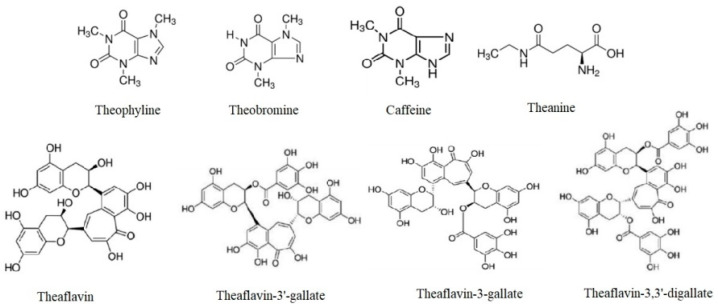
Structure of tea methylxanthines, theanine and major theaflavins.

Among the many chemical substances in tea shoots, the high concentrations of polyphenols and caffeine are particularly notable. Proteins, carbohydrates, amino acids, lipids, caffeine, polyphenols, fiber, and minerals are the primary chemical components of the green leaf [39]. Tea leaves contain chemical constituents, including methylxanthines, vitamins, and more than 600 volatile compounds. It comprises 25 to 35% polyphenols by dry weight [8]. The most significant antioxidants, catechins, make up 25% of their dry weight [12,40,41]. Besides theanine, fresh tea leaves include methylxanthines (theophylline, caffeine, and theobromine) [29,42], and the main TFs include theaflavin, 3-gallate, 3′-gallate, and 3,3′-gallate (Figure 2) [43]. Sugars, amino acids, organic acids, polyphenols, caffeine, and volatile flavor molecules affect tea’s taste, color, flavor, brightness, smell, and astringency [44].

## 3. Black Tea’s Manufacturing Cycle and Its Influence on Quality Characteristics

**Figure 3:**
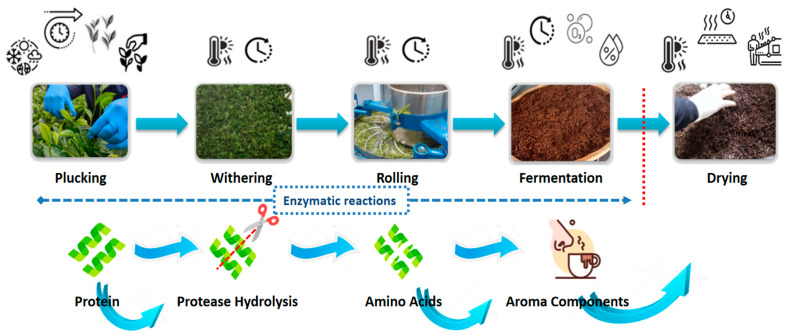
Factors affecting black tea quality and changes that occur in chemical composition during processing.

Black tea’s quality highly relies on the physicochemical processes involved in its production. Soon after tea leaves are plucked, they undergo various processing steps, including withering, rolling, fermentation, drying, sorting, and storage [45,46,47]. Figure 3 shows different processing steps involved in the manufacturing of black tea and factors influencing the quality of black tea during processing and storage. The quality of the final product is determined by a series of chemical reactions at each stage [48].

### 3.1. Plucking/Harvesting/Picking

Plucking, often known as picking, is identical to harvesting for all other crops [47,49]. Black tea leaves can currently be picked using a variety of techniques. The three primary techniques for plucking tea leaves are manual plucking, knife, and machine plucking. Mostly, black tea is hand-picked. The plucking method, standard, interval, and plucking season greatly influence the quality of the final output [50].

#### 3.1.1. Plucking Method

Tea leaves are manually plucked by tea pluckers [49]. Long-term tea producers developed a tiger’s mouth plucking technique ideal for picking green leaves. When choosing fresh leaves, the thumb and fingers are primarily separated, and the bud tip is inserted from the center [51], successfully avoiding crushing and scalding by holding them all in the palm. In particular, the machine and knife picking approach is unable to achieve selective picking and cannot guarantee that fresh tea leaves quality and the size of chosen leaves are essentially the same. However, the benefit is that it can increase picking productivity and lower manual picking costs, which is advantageous for low-grade, mass-produced tea [52]. Black tea harvesting is conducted primarily by hand and has not been mechanized. Therefore, the correct harvesting of fresh leaves must be performed manually; however, there are many obstacles, such as a lack of knowledge, improper technical practices among tea producers, and inefficiency. During crown cultivation, the new shoots of tea trees are damaged, the old and tender ones are uneven, and the leaves and branches are severely damaged and mixed up, which not only affects the growth of tea trees but also quality improvement. As a result, technologies for tea picking and cultivation are balanced. Pinch plucking, used for soft tea specifically, requires less time and is less efficient than other methods. Picking with both hands is an efficient manual picking technique that demands quick and stable technology and prevents leaves from being lost or damaged. Manual picking also involves moderately picking high-quality fresh leaves [51]. A remarkable difference was found between hand- and shear-plucked tea. Table 1 summarizes the alterations in the qualitative characteristics of black tea as a result of shear plucking. Shearing reduces TF, a quality factor. While shearing, highly polymerized substances and TRs are not acceptable above a certain level, while the total liquor reduces slightly, which tasters noticed. Shearing decreases water extract, which impacts cuppage.

Shearing reduced the crude fiber content, an unfavorable parameter whose maximum value was set at roughly 16%. Professional tasters rated hand-plucked tea higher than shear-plucked. They noted that hand-plucked tea had a better flavor and color than shear-harvested tea. After considering all factors, shear harvesting reduced production costs. Shears lengthened the interval between plucking and decreased productivity or yield. Furthermore, plucking by hand yielded finer leaves compared to shearing; shearing yielded coarser, more mechanically damaged leaves; whereas hand-plucked tea contained just “three leaves and a bud”. Non-selective shear plucking removes each accessible shoot, although immature shoots have the potential to transform into a new batch of shoots in a short time. Shear harvesting reduced total catechins and polyphenols significantly. This shows a decrease in made tea quality. Mechanization boosted chlorophylls but decreased carotenoids. Shearing boosted lipoxygenase activity but decreased polyphenol oxidase. Except for linolenic acid, which remained nearly constant, saturated fatty acids declined while unsaturated fatty acids rose [53].

The plucking method affected black tea’s sensory and chemical qualities. Plucking with hands exhibited more brightness, TF, volatile flavor compounds (Group II), flavor index, caffeine, and sensory attributes as compared to shearing, regardless of variety [50]. Tea harvesting is the most expensive agricultural process. It is delicately balanced. Without compromising bush health or product quality, it should maximize shoot production. The harvesting technique should be the best possible balance of quality, yield, and cost. Shears require long plucking intervals (24 rounds per year), whereas hand plucking requires short intervals (32 rounds per year). Short plucking intervals yielded the most; therefore, hand plucking was best. However, shear harvesting is cheaper.

#### 3.1.2. Plucking Standard

**Figure 4:**
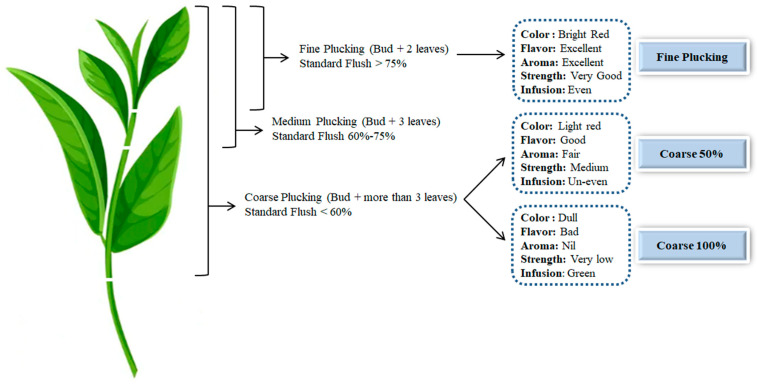
Percentage of standard flush for standard leaf plucking and effect of fine and coarse plucking on black tea’s quality.

Plucking standards are essential factors in determining black tea’s quality. The standard of plucking has a significant impact on tea quality and yield. Many high-quality teas require uniform and tender leaves. Plucking standards are often classified as fine, medium, or coarse, with the given percentages of 75%, 60–70%, or less than 60%, respectively (Figure 4). Plucking with 25% coarse and 75% fine leaves balances yield and quality. Coarse leaves and immature shoots are undesirable because standard flush was dependent on standard leaves [47,54,55].

Good crush tear and curl production requires high-quality plucking, which is important not only for the tea liquor’s quality but also to keep the crush, tea, and curl machine from blunting by cutting woody material, stalk, coarse leaf, and branch. To balance black tea quality while ensuring the plucker’s productivity is not compromised, it has been suggested to pluck a bud and two leaves [12,47]. Furthermore, only healthy tea shoots should be plucked. Care must be taken during tea plucking to ensure that only healthy tea shoots are harvested [56].

Fine plucking removes a bud and two leaves, while coarse plucking removes a bud and three to four leaves [57]. In all tea cultivars, a bud and two leaves’ plucking standards are considered to be an ideal compromise among plucker productivity, quality, and yield. Therefore, regardless of the manufacturing process or tea leaf variety, most tea processing industries comply with this plucking standard [47,58]. However, to achieve more outstanding biomass production in a single plucking round, some producers opt to use less delicate shoots. However, the coarse plucking standard reduces plucking frequency because new shoots need more time to mature. Cumulative biomass production may not be beneficial over time [59,60]. In contrast, the study found that fine plucking boosted yields when plucking rounds were shorter. As a result, fine plucking at shorter intervals is suggested to increase yields unless labor is a limiting factor [47,61,62]. Indeed, fine plucking young shoots increases yields [63]. In milky and plain tea, fine plucking produces a bright red color (Figure 4). Compared to coarse plucking, the flavor and smell were pleasant [64]. Similar results were found by [58], who discovered that fine plucking is of high quality. With coarse plucking standards, black tea’s total water-soluble solids, caffeine, TF, and total ash decreased, whereas crude fiber, TRs, and florid increased [60].

Coarse plucking reduces black tea’s plain and aroma quality [47]. The findings were attributed to the greater levels of polyphenols present in young tea shoots [58], making tea beverages’ plain quality characteristics decline as leaves older. So, the catechins (flavan-3-ols) responsible for making TFs and TRs, which are black tea’s quality parameters, are reduced [58]. But as the leaves age, the amount of chlorophyll, which lowers black tea’s quality, increases [60,65]. Plucking standards varied widely, as according to [60,66], fine plucking exhibited massive water-soluble solids, caffeine, and TF content but low crude fiber and ash. Catechin, polyphenol oxidase activity, and TFs were maximum in a bud and two leaves. Because mature shoots have more polyphenol oxidase, coarse plucking produces high TRs and low TF. However, plucking a bud and leaf and a bud provided tea with high quality with highly volatile flavor compounds (Group II), caffeine, total water-soluble solids, flavor index, crude fiber, and TFs (Table 2).

The plucking standard also results in the variation of catechin level. When plucking was coarser, catechin levels decreased. Each plucking standard changed catechin concentration. Even though epigallocatechin gallate was the most prominent and (+)-catechin the least prominent flavanol, the decline in individual catechins varied with the plucking standard. Due to plucking standards, epigallocatechin gallate and epigallocatechin exhibited the most variation, whereas (+)-catechin exhibited the least [61]. Caffeine and polyphenols, which are plentiful in the bud and decrease with leaf coarseness, are the tea shoot’s primary components [12,47]. Flavon-3-ols catechins are plentiful polyphenols in young tea shoots and are essential for black tea production. The primary catechins found in tea leaves are catechin, epicatechin, epicatechin gallate, epigallocatechin, and gallocatechin [13,40].

Black tea made from mature tea shoot leaves is far lower in quality. Black-tea-producing countries set a standard of plucking buds and two leaves to profit economically. This produces high-quality teas. Picking a bud and two leaves offers quality and yield.

#### 3.1.3. Shooting Period/Plucking Interval

Plucking intervals affect black tea’s quality, green leaf standard, and chemical composition. Long intervals between plucking contribute to inferior leaf quality with low TFs and more mature leaves, the total amount of Group II volatile flavor compounds, level of caffeine, and black tea’s taster ratings. However, the total of Group I volatile flavor compounds—which give tea its poor, grassy, green flavor—increases with longer intervals between pluckings [67]. The decrease was primarily due to an unsaturated fatty acid increase [69], leading to a rise in black tea’s Group 1 VFC [67]. So, plucking with short intervals increases crop quality and yield. The plucking interval can affect shoot distribution, crop quality, and quantity [70]. A short plucking round for black tea was preferred over long intervals because it had higher caffeine, chemical aroma quality indices, brightness, TF, and sensory ratings. Plucking with long intervals produces coarser leaves and reduces tea yields as compared to short plucking intervals [50]. Shorter plucking intervals improved quality by plucking less coarse leaves. Frequent plucking intervals result in better yields due to an increase in auxiliary bud development rate because of frequent reduction in apical dominance by apical shoot decapitation [60]. In Malawi, increasing the plucking interval enhanced tea yield. Shorter plucking intervals resulted in more fine leaves with a bud and two leaves than more extended intervals. As the plucking rounds prolonged, the percentage of one leaf and a bud and two leaves and a bud decreased, whereas the opposite was found for three leaves and a bud and four leaves and a bud [67]. In the Assam Valley of Northeast India, shoots are plucked at seven-day intervals when the chemical components required for a good cup of tea are at their peak. Any change in the plucking length (time period) for shoot biomass development may affect the leaf physiology, dynamic metabolic system, and various chemical compounds liable for black tea flavor [71,72]. Table 2 exhibits the chemical composition of the four samples with varying plucking intervals. As leaves mature, total water-soluble solids decrease while crude fiber increases. Due to lower ash and fiber content, teas with a five-day plucking interval are regarded as good organoleptically, while black teas with a seven-day plucking round contain a balance of soluble solids, volatile flavor compounds, caffeine, and ash, so they are considered good by tea tasters. Teas manufactured from nine- and eleven-day plucking intervals are considered inferior in quality to five and seven days due to a loss in soluble solids and a huge rise in crude fiber. From days five to eleven of the plucking interval, TFs and TRs, black tea’s most characteristic compounds, vary with leaf maturity [60,71].

It has been reported that brisk, bright, and colored teas have been highly valued for caffeine, TF, and thearubigins content. Shorter plucking intervals resulted in more excellent caffeine and TF content in teas. With longer plucking rounds, these values declined. Insignificant changes in TRs were found. While Tasters A and B gave the teas no order, Taster C gave them a systematic drop as the plucking rounds increased. Taster C found that plucking with short intervals resulted in teas with excellent ratings [67].

The Group I volatile flavor compounds contribute a lower flavor to the tea’s quality; consequently, as the picking durations were reduced, the tea’s flavor enhanced. However, as plucking rounds were longer, the sum of Group II volatile flavor compounds declined. Although the VFC of Groups I and II rose and declined, respectively, with an increase in plucking length periods, Even though the plucking length rounds changed, the total volatile flavor compounds did not vary. However, flavor index is the most accurate measure of tea flavor quality and it is decreased with longer plucking rounds [66,73]. These results are consistent with those reported by [71] but for shorter plucking intervals. Tea quality measured by flavor index, caffeine, TF, volatile flavor compounds, and evaluation by tea tasters were found to be decreased with extended plucking.

It has been observed that shortened plucking intervals result in greater yields and a more significant percentage of a bud and two leaves in the harvested shoots compared to long picking intervals. Shortening plucking intervals can maximize yields and tea quality [70]. If plucking is not selective, longer plucking intervals produce shoots with more than two leaves and a bud (coarse leaf) compared to short plucking intervals. Hence, prolonged plucking rounds that many tea growers currently adopt result in decreased quality and yield.

#### 3.1.4. Plucking Season

The seasonal changes in temperature and weather, humidity, rainfall, and soil water deficiencies affect annual yield as well as annual yield distribution [74,75] and also black tea’s quality [76]. The performance of most crops, including tea, varies from season to season and from locality to locality [77]. These variances result from a difference in growth parameters [78], which causes a change in the overall quality and chemical composition of black tea [79]. Tea quality depends upon seasonal variations in terms of moisture content, TFs, and TRs, which also link well with tea color [80]. TFs and TRs, two significant polyphenols used as black tea quality indicators, make up 50–70% of the phenolic components in tea water extract [81]. Black tea’s quality is mostly determined by the phenolic chemicals that are found in young tea shoots [82]. Low-quality black teas have a low total polyphenol concentration [83]. Consequently, levels of total polyphenols are essential to black tea’s quality, and these concentrations are influenced by their concentrations in fresh tea shoots [60,84].

It has been examined previously how harvesting seasons affected the physicochemical properties of “Yinghong 9” (Yh) and its mutant “Huangyu” (Hy) black tea with large leaves. The results showed spring through summer-processed black tea showed significant soluble sugar and caffeine increases, while free amino acids decreased significantly. This variation was noticed in both Yh and Hy [85], and the outcomes follow previous findings [86], which defend it from UV radiation across the three harvesting seasons [87]. Summer and autumn showed a decrease in TFs and TRs, whereas the TF-to-TR ratio seems to be enhanced for both Hy and Yh. These results are in accordance with [88,89], which found that infusion color and TF content varied with variation in harvesting season.

Tea is cultivated from the equator to the subtropics, where seasonal changes may be extreme [74,90]. Seasonal temperatures in the Kericho District, Kenya, range from 15 to 17 °C compared to the Tea Research Foundation of Central Africa at 18–24 °C [91]. Early research in Central Africa found that fresh apical shoots have the highest level of flavanols in the winter season. Tea shoots harvested under conditions of slow growth, such as during the cool season, exhibited higher levels of simple catechins compared to catechin gallates, and the most significantly affected compound is epigallocatechin gallate (EGC). Contrarily, total flavanol concentration is highest during the summer (growing season) and lowest in late autumn in the northern hemisphere. Cool, slow-growing seasons result in black teas with high quality but low production [92]. Dry or severely cold seasons reduce yields [93], which is in accordance with a previous study [94] that declared that slow shoot growth in dry and cool seasons increases black tea quality, while fast tea flush growth in wet seasons, especially rainy periods, lowers black tea’s quality. If the soil is moist, high temperatures promote rapid shoot growth, increasing production but reducing black tea quality. In contrast, cold weather slows shoot growth, reducing yields but improving black tea quality. Dry weather, desiccating breezes, and cold nights favor flavor compound biogenesis, producing flavorful black teas. Thus, slow-growing shoot teas are better quality and more valuable [74,95], whereas warm, wet seasons result in faster growth and provide high-yield but low-quality black teas [60,96]. Cactines gallates (CGs) to cactines (Cs) ratios vary seasonally for the same variety, var. assamica, with warmer months having greater ratios and cooler months having lower ratios [92].

The variations resulting from picking seasons over chemical constituents like TFs, TRs, and moisture content show that the content of TFs and TRs are highest in early flush (March) and lowest in rainy flush (July) but moderate in backend flush (November) tea samples. As quality grade decreased, TF and TR contents dropped in each of the seasonal samples. The TR/TF ratio was 8–10, which is ordinary for well-fermented tea. The moisture content did not show any significant difference in all tea samples due to modern manufacturing methods. Tea samples from March correlate positively with TF% and TRs% but negatively with moisture content. Because fresh tea shoots were abundant in catechins and polyphenols, they decreased over-harvesting seasons, lowering tea leaf TFs and TRs. July samples positively correlated with moisture content but negatively with TF% and TRs% because of slight moisture involved in the manufacturing process of black tea due to the rainy season. November samples have smaller TFs and TRs values compared to March samples but higher than July samples, indicating a poor correlation with TF%, TR%, and moisture content [80]. The primary flavanols in tea shoots for both assamica and sinensis varieties were epigallocatechin, epicatechin gallate, and epigallocatechin gallate, having epigallocatechin gallate as predominating. Epigallocatechin was more excellent in spring as compared to summer, but epicatechin gallate and epigallocatechin gallate were higher in summer. Additionally, epicatechin gallate and epigallocatechin gallate were greater in tender and younger shoots, while epigallocatechin was more prevalent in peak matured shoots [92]. These variations in flavanol contents appear to be the primary factor affecting tea quality [97].

Thus, information on the levels of epigallocatechin gallate, epicatechin gallate, and epigallocatechin in fresh Australian tea green shoots could be utilized as a sign of seasonal fluctuations in black tea’s resulting quality. TF, the primary polyphenol in black tea, provides black tea with its distinguishing sensory properties, i.e., taste and color. The total TF amount in the resulting black tea and the amount of epigallocatechin in the fresh shoots are highly correlated [92]. Consequently, correlations between the quality of tea samples and chemical constituents based on distinct seasons are of the utmost importance for auction centers and the tea industry. As a result, the plucking season should be prioritized while manufacturing black tea.

## 4. Withering Stage

Withering, as the initial step in manufacturing black tea, is crucial to produce high-quality black tea. Tea processing begins with withering, where freshly harvested tea leaves are spread out in a proper withering system to lose moisture before processing [49]. For the subsequent steps, tea leaves go through a series of physical and chemical changes [47,98]. In tea processing, leaf moisture is crucial; tea leaf withering partially dries the surface and core moisture of tea leaves [99]. Black tea leaves are usually withered at room temperature to reduce moisture. Tea shoots undergo withering from being harvested until they are macerated or rolled [47]. Turgid tea shoots become flaccid as moisture levels decrease on a wet basis from ~70–80% to 60–70% during the withering process [100]. As green leaves wither, their moisture content drops to 60–70%, as noticed by [101,102]. Tea leaves are more flexible after withering and can be rolled and twisted without breaking. Manually handling green tea leaves during ordinary withering damages fresh tea leaves, lowering the final quality of black tea [103]. Changes in biochemical compounds in tea shoots during withering affect quality. Properly withered tea leaves improve color, aroma, flavor, and other qualities, so tea leaves must undergo proper and even withering to obtain high-quality tea.

Chemical and physical withering are the two main kinds of withering. During the procedure, tea shoots experience chemical and physical changes [47]. Immediately after harvesting tea leaves, chemical withering occurs. This process breaks down complex chemical compounds into volatile flavor compounds, simple sugar, and amino acids. The enzymatic activities and caffeine content rise with withering. Additionally, it has been found that during the process of withering, lipids degrade into simpler molecules, and the catechin amount drops [100]. The withering phase’s dehydration shock induces enzymatic ripening and gives the shoots a floral flavor. On the other hand, physical withering is the process of moisture removal from tea leaves and changing tea leaves’ cell membrane permeability. Both methods of withering are essential, and it is difficult to achieve ideal attributes (color, flavor, aroma, and taste) from uneven or un-withered leaves. Moreover, proper and even withering seems to be the most crucial step for the rest of the tea-making process, as it improves taste, flavor, and other qualities [49,104]. Compared to chemical withering, physical withering requires less time [47]. Physical withering is primarily affected by relative humidity, temperature, and time [44].

**Figure 5:**
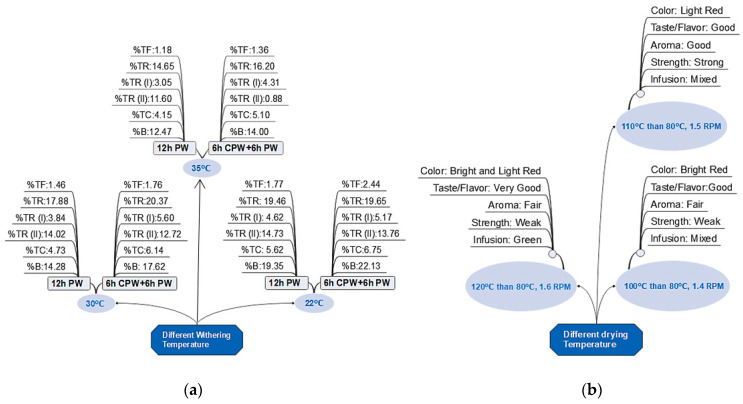
(**a**) Effect of withering temperature on black tea final quality. (**b**) Effect of drying temperature on black tea final quality. TF = theaflavin, TR = thearubigin, TC = total color, B = brightness, RPM = revolutions per minute, CPW = initially kept in 100% humid conditions for 6 h followed by physical withering 6 h, PW = only physical withering for 6 h.

Another major determinant of tea quality is withering temperature. Excessive heat during withering destroyed the leaf cell matrix, resulting in earlier uncontrolled fermentation-like responses [60]. It has been evaluated how temperature and moisture loss affect TFs, TRs, and volatile flavor components development during withering. Results show that restricting moisture loss during the early withering stage improves black tea quality. Additionally, low withering temperatures produced brighter teas with more TF and TR content. It has been found that catechin degradation was low after a few hours of moisture loss restriction. Catechin concentration decreases to a minimum after prolonged moisture restriction. It has been concluded that the rate of catechin breakdown was highest in normal physical withering without leaf storage. Reducing the traditional withering time from 12 h to 4 h in a modified approach resulted in various desirable changes in biochemical compounds, which improved tea quality and brightness, so it has been concluded that TFs and brightness improved with the modified withering procedure [105] (Figure 5a), increased TFs is crucial for brighter tea infusion [106]. Here, it was accomplished due to a reduction in catechin oxidative degradation in W1 (kept at 100% humid conditions initially for four hours then physically withered for eight hours and W2 (kept for eight hours in 100% humid conditions initially then physically withered for four hours), shoots had been stored initially at 100% humidity before accelerated moisture loss during normal withering. It has been concluded that leaf storage is necessary for quality enhancement. Processing of black tea, especially whilst withering, triggers endogenous enzymes (PPO and POD) to oxidize and dimerize monomers into dimeric compounds of TFs [28].

Some unfavorable enzymatic reactions occur at high temperatures, leading to an undesirable amount of TFs and TRs, which results in an increase or decrease in the flavor index, brightness, and black tea’s sensory quality. The two primary kinds of VFC are E-2-hexenal and hexanal (Group I) and geraniol and linalool (Group II). Group II volatile flavor compounds contribute to a good effect on aroma, and Group I volatile flavor compounds grant inferior aroma [83]. Withering decreases the total amount of Group I volatile flavor compounds while increasing the total of Group II volatile flavors [107,108]. The Group II VFC was raised when tea shoots were restricted in their moisture loss for a prolonged period prior to physical wither, which improves the aroma’s overall quality. However, stressed shoots have the most Group I volatile component. The flavor index needs to be optimal to improve quality. It has also been concluded that a low temperature of 22 °C increased TF, total color, and brightness [105]. The over-withered tea shoots harden and blunt the roller or maceration machine. As a result, tea fiber content rises, as do the possibilities of over-firing and over-fermentation. In contrast, a high content of moisture in withered leaves leads to clog crush, tea, and curl (CTC) and the Rotorvane machine during operation [109]. Withering produces high-molecular phenol oxidase, which is essential for fermentation [102].

It has been examined how withering affects fermentation [110]. During fermentation, (PPO) polyphenol oxidase activity affects TF and TR production. TFs and TRs give tea liquors briskness, brightness, and “body”. It has also been found that more extreme withering and greater moisture loss decrease PPO activity. Therefore, black tea loses briskness and brightness. Tea leaves, which are shortly withered, result in more briskness and brightness due to a rise in TF formation. Extreme moisture loss, such as long duration or high temperature, reduces green leaf PPO activity during withering [41]. It has been reported optimum withering time is 14 h [110]. Some researchers suggested 16 h [85], whereas some reported that the withering time should be 17.5 h [111]. It has also been suggested that the withering time should be limited to 18 h [102]. Some researchers carried out withering for 20 h at ambient room temperature [112]. According to one study, black tea quality degrades after 20 h of withering [113]. It has been reported that there is no set time for withering; however, 14 h to 18 h is considered as the optimum duration [100]. Withering duration affects black tea quality. The color of tea liquor and TR content increased in 16-hour-withered leaves. The most colored tea beverage came from 16-hour-withered leaves and also possessed excellent sensory ratings [98], which are consistent with [53], who reported that withering improves tea aroma. Extended chemical withering improved black tea taste quality. While withering time did not affect tea liquor brightness or TF content, 8 h withering produced black tea with low biochemical and sensory quality [105]. These outcomes are in accordance with [114], who stated that briskness and brightness result from TFs; thus, withering did not significantly affect TF content. In conclusion, withering time did not significantly affect the brightness of tea liquor. Conversely, it has been found that as withering length increased, TF levels declined; thus, liquor would be less bright with longer withering duration [44] The rainy season teas had the lowest TFs and brightness, probably caused by uneven and low withering, which reduces PPO activity [88]. However, Ref. [13] compared the fermentability of freeze and normally withered leaves. Following a four-hour freeze, withered leaves had greater TF levels and brighter liquid. This approach shortens withering time. As withering progressed, oxidation reduced TFs to generate a red-brown pigment called TR. After that, TRs reacted with amino acids, producing a dark brown compound known as theabrownin [115]. The transformation of TFs to TRs may account for its decreasing trend during withering [45]. These findings are consistent with a prior study demonstrating that TFs decrease as moisture content decreases during withering [44]. Withering involves a rise in soluble proteins, amino acids, and cell membrane permeability and a decrease in protein [105].

It has been shown that the duration of withering has a direct effect on volatile compounds content. The leaves following hard withering have more hexenal, linalool, and oxides, which explains why such tea fragrances more [116]. Withering temperature affects the brightness of black tea, and withering at low temperatures can result in brighter tea [105].

Allowing withering for 8 ± 0.5 h to 10 ± 0.5 h can enhance quality and nutritional value. It reveals that withering time duration is essential for biochemical properties. This stage can be controlled and monitored to produce high-quality black tea [45].

Good-quality black tea requires optimized chemical withering. There is no set standard for withering. It varies with leaf quality, requirements, and ambient conditions. For maceration, withered leaves should have 68–72% moisture is recommended. Tea shoots to wither properly depends on plucking, transportation, time, temperature, and environmental conditions. Thus, withering must be regulated to ensure high-quality tea [100]. A previous study concluded that air temperature, velocity, and humidity were regulated in tailored troughs to complete withering with desired physical and biochemical qualities in a short time in a tailored trough compared to natural withering [117]. Over-withering should be avoided because it impairs the enzymatic processes that produce black tea’s quality [118]. Tea shoots undergo metabolic changes during withering, which affect tea quality. Tea leaves must be adequately withered to achieve the desired aroma, flavor, color, and overall final quality.

## 5. Rolling/Maceration Stage

Post-withering tea leaves are macerated or rolled to rupture plant cell structures, allowing catechins, polyphenol oxidase, and peroxidase to interact during fermentation [97]. Rolling extracts and twists leaf juice [54]. The main goal of rolling is size reduction and cell destruction to expose new surfaces to air during fermentation. It also presses out juice and coats leaf particles with a thin juice coating to accelerate chemical reactions [119]. This process is crucial for black tea production since most TFs are generated during rolling [112]. During rolling, enzymes are released from the leaf when it is broken and exposed to oxidation. Crush particles (dholes) were crushed by the rotor vane machine after rolling. After crushing, the material was run through a CTC crush tear and curl machine to make particles finer. The material then experiences a roll breaker, which breaks the twisted balls and reduces fermentation [60]. In Sri Lankan black tea production, orthodox rolling is used to gently roll tea leaves. The rolling method produces black orthodox tea. For tea leaf pulverization in Central Africa, a Lawrie tea processor is used, which can also be used to treat them harsher [120], whereas Indian and Kenyan tea leaves are crushed and macerated using crush tear and curl machines [120,121], which are referred to as black crush tear and curled teas. The maceration methods that influence black tea’s chemical composition and quality were investigated by [113]. While rolling tea shoots, polyphenol oxidase oxidizes catechins and leads to the formation of TFs and TRs. TFs provide tea liquor’s brightness and briskness, while thearubigins contribute to taste and orange-brown color [122]. With an exhaust temperature below 49 °C for a long time, the post-fermentation process will soften the liquor. Exhaust temperatures above 57.2 °C accelerate moisture removal, resulting in hardened tea with hard particles but incomplete drying. Such types of tea do not store well and have harsh liquors [119]. Catechins and gallates oxidize to TFs and TRs. Black tea’s TFs and TRs not only rely on the green leaf’s oxidase activity, the content of flavanol and protein but also the processing method and leaf resistance to mechanical damage. This resistance affects CTC processing less than orthodox. Crush, tear, and curl and orthodox teas differ greatly in TF and TR content. TFs and TRs are higher in crush tear and curl teas compared to orthodox teas. [123]. These two processing methods have less effect on TRs than on TF. The impact on flavonoids is associated with the variation in the concentration of volatile compounds generated by each distinct processing technique [124]. This may explain why CTC tea was less aromatic than other teas, as it had less volatile compounds with floral notes like linalool and its oxides than orthodox black teas [125]. Rolling speeds (35, 45, 55, 60 r/min) affected gongfu black tea flavor and composition. Electronic tongue and color difference examinations showed that Congou black tea had the best taste and aroma at 45 r/min. Rolling speed affected tea pigments (TFs, TRs, and theabrownins), phenolic and organic acids, but not other metabolites. Carbohydrates, quality index TRs, and aspartic acid were found to be highest in Congou black tea prepared at 45 r/min, while theabrownins and organic and phenolic acids were lowest, indicating that this rolling speed was better for flavor formation. It also showed that rolling times affect black tea quality [126]. Rolling times (50 min, 75 min, 100 min, 125 min) also affected black tea’s chemical composition and sensory quality. TFs, TRs, theabrownins, and the dominant TF contents significantly differed over four rolling times of Congou black tea [68], as shown in Table 2. Prolonged rolling times enhanced tea liquor color and pigments, i.e., TFs, TRs, and theabrownins. The maximum umami and lowest bitter intensities and higher index of quality (10 TFs + TRs)/TBs were achieved with 100-min-rolled black tea. Those findings revealed that rolling times affected amino acid oxidation, catechin oxidative polymerization, and flavonol O-glycoside hydrolysis.

A proper rolling time of 100 min made a superior quality of Congou black tea, while it has also been found that rolling for 25 min reveals good results lasting less or more than that time [64]. Processing stages and rolling methods also affect black tea caffeine and theanine levels. Orthodox and Cay–Kur procedures reduced theanine content from 10.0–3.42 to 7.73–3.97 mg g^−1^ dw in all processing phases, respectively. Rotorvane techniques reduced theanine loss [127]. Rolling temperature affects Congou black tea taste. Congou black tea tastes better at a rolling temperature below 25 °C and best at 16 °C. Low or intermediate rolling temperature (10–25 °C) produced better-quality tea infusion based on taste component analysis [128]. One study evaluated the rolling temperature effect on Congou black tea’s physicochemical, sensory quality, and aromatic compounds. A low rolling temperature of around (20 ± 2 °C) maintains potent polyphenol oxidase and peroxidase activities, significantly increases TF content, and enhances tea liquor’s brightness and redness value [129].

The caffeine and polyphenols content declined significantly, whereas aldehydes, ketones, alcohols, and other aromatic compounds increased. Thus, rolling Congou black tea at lower temperatures is a novel way to improve its quality. The black tea polysaccharide yields for rolling times of 0 h, 0.5 h, 1 h, 1.5 h, and 2 h were 1.17%, 0.99%, 1.07%, 1.26%, and 1.21%, respectively, with 1.5 h rolling producing the highest. A rolling time of 1.5 h drastically reduces particle size and molecular weight, and stability is most remarkable [130].

## 6. Fermentation/Oxidation Stage

After leaf disruption, fermentation is another crucial step in black tea production due to chemical changes [131]. Therefore, fermentation is the most crucial factor in the determination of the processed black tea’s quality [12,132]; oxidation of catechins during fermentation leads to the formation of TFs and TRs [133] liable for briskness, brightness, color, strength, and black tea liquor [134]. In pairings, catechins generate different TF compositions [12,107,135]. In black tea leaves, enzymes oxidize and partially polymerize around 75% of the catechins [136]. Polyphenol oxidase and peroxidase oxidized catechins [106]. When catechins are exposed to oxygen, these enzymes develop oxidized polyphenolic substances like TFs and TRs [40,137,138]. The fermentation environment’s temperatures, relative humidity, time, pH, and oxygen affect these compounds greatly. When black tea ferments properly, it turns green to coppery brown. It also smells fruity, brisk, rich in flavor, and tastes strong. Fermentation needs a set temperature, humidity, and time. During fermentation, good aeration increases TFs and TRs, whereas less aeration results in a reduction of those compounds. In the same way, high temperatures also reduce TF formation [49,139].

### 6.1. The Influence of Fermentation Process Parameters on Black Tea’s Quality Attributes

Postharvest techniques and raw materials determine tea liquor quality. High-quality black tea has a rich flavor, brisk, brighter reddish-brown color, and strong taste. Fermentation conditions like time, temperature, relative humidity, and oxygen affect these quality attributes [140]. Thus, fermentation parameters must be controlled to make high-quality black tea liquor [141].

### 6.2. Time

The fermentation period greatly affects black tea quality [13] (Table 3). There is no set amount of time it relies on plucking standard, degree of rolling or maceration, type of tea, and degree of withering. Liquor quality attributes include brightness, astringency, strength, and briskness peak at different times. Thus, to achieve the optimal result, process parameters must be optimized [107]. In general, fermentation is carried out at around 20–30 °C for 30–120 min, although 25 °C for 60 min is considered the optimum [40,142].

**Figure 6:**
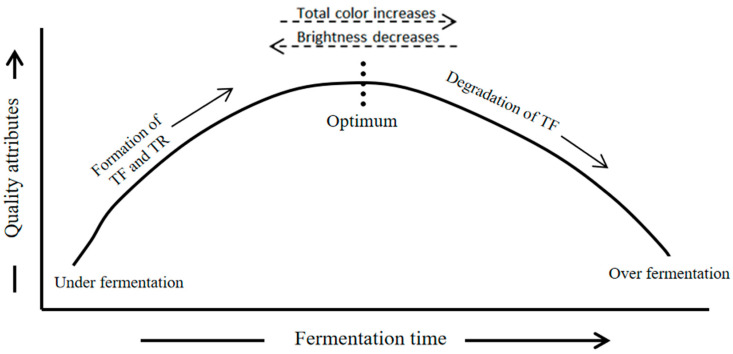
Variation in quality characteristics of black tea during fermentation.

The quality of the prepared tea depends on when the fermentation process is stopped [13]. As shown in (Figure 6) as fermentation time increases, TFs and TRs concentrations and desirable quality features approach optimum levels and then degrade if prolonged [12,107,143]. An astringent, brisk taste and golden color come from TF, while a brown-red color and rich mouth feel are contributed by TRs [144]. Crush tear curled and orthodox teas ferment for about 55–110 min and 2–4 h, respectively [145]. Fermentation time significantly affects the content and changes of tannins, TF, TF/TR characteristics, and brightness, which rely on the genetic potential of plants [146].

TFs degrade to TRs and thicken tea liquor if fermentation is prolonged [147,148]. Despite its body, over-fermented liquid lacks quality. For optimal results, maintain the (TF:TR) 1:10 ratio to achieve the overall best result [12,148]. For fermentation at 20 °C, total TFs, total TRs, total color, brightness, and briskness peak at fermentation durations of 90 min, 120 min, 120 min, 60 min, and 60 min are in the given order [140]. To oxidize catechins and obtain the appropriate TF content, macerated tea leaves are fermented shorter than rolled tea leaves. Normally, fermentation takes 45 min–3 h at room temperature [121]. At 45 min of fermentation, the TF formation curve peaked [13]. However, after 110 min, it fluctuated because of changes in polyphenol oxidase activity, but according to [142], fermentation at an ambient temperature of 25 °C for a shorter duration of 60 min increased TF formation. As a result, the black tea produced is astringent, brisk, bright, and probably more favorable to human health. TFs and TRs improve the liquor brightness prediction and overall color in black tea compared to using either alone. TFs had more briskness and astringency than TRs. TFs predicted them best. Taster brightness evaluation was best predicted or linked with TRs.

Increasing black tea fermentation time decreases TFs and brightness but increases total color and TRs [44] (Figure 6). Fermentation carried out at 25 °C for 60 min results in more TRs and total color and favors dark-colored thicker black tea [142]. During early fermentation, TR formation increased as fermentation progressed, and TRs peaked and then slowly dropped [149]. The best results were obtained with increased fermentation time [64] (Table 3). These findings agree with those of [61], who found that a rising fermentation trend was most productive.

The optimal fermentation time for Yunnan Congou black tea was 3 h, and that fermentation duration significantly affected tea quality. The formation, retention of TFs, TRs, and quality index were all high. The 3 h fermented tea samples had a higher total aroma, more significant flavonoid glycoside degradation, and higher soluble sugar component formation and transformation. The tea also had stronger taste attributes [150]. High temperature favors TR production, suggesting that peroxidase may be most active at high temperatures. At a lower temperature of 20 °C, catechins convert more efficiently to TFs. Catechins also convert to TRs at this temperature, although fermentation takes longer [40]. A suitable fermentation time for Yunnan Congou black tea YCBT 3.5–4.5 h was the best to obtain bright red tea liquor [151]. A longer fermentation time produces large amounts of TRs and theabrownins but not in favor of liquor brightness [152]. Fermentation is a very critical step because it determines all the quality characteristics, i.e., color, flavor, aroma, strength, etc., so it must be given due attention during tea processing.

### 6.3. Temperature

The temperature involved in fermentation significantly affects the enzymatic activities and, consequently, the process of fermentation. A low or high fermentation temperature can inactivate enzymes and stop enzymatic processes. Enzymes are protein in nature and high temperature leads to denaturing [153]. Enzymes (PPO and PO) break down tea leaf chlorophyll during fermentation. Superior quality tea requires a controlled temperature during the process of fermentation. A 20–35 °C air temperature during the process of fermentation affected crush tear and curl CTC black tea quality, resulting in peak TFs, TRs, ratio, and brightness at 20 °C [40]. Fermentation at 25 °C for 60 min is considered best for Chinese and promising 100 cultivars [142]. Generally, 24–27 °C temperature is considered best for fermentation, but different kinds of teas have different optimum conditions [145]. On the other hand, [107] suggested 27–29 °C temperature and a time range of 2 h 30 min to 3 h 45 min or 55–110 min for orthodox tea or crush tear and curl black tea, respectively. PPO and PO enzymes are most active at this temperature, resulting in good-quality tea. Different clones of macerated tea leaves (dhool) fermented for 0–180 min at 15–30 °C, and fermentation at 20 °C resulted in the highest black-quality tea for all clones. Low-temperature fermentation creates good-quality black tea, while long fermentation durations and high temperatures produce intense color and high TF content [154]. Teas with high fermentation temperatures have higher color and TR values but lower sensory values, brightness, and TF content [96]. Due to its more robust taste and aroma, at 28 °C, fermented black tea has the highest sensory ratings. Additionally, 28 °C fermentation temperature makes black tea more effective at inhibiting intestinal glucose uptake, oxidative stress, and glycosidase activities. Altogether, results show that lowering the fermentation temperature could enhance the bioactivities and sensory qualities of black tea [155]. Low fermentation temperatures assist in the retention of the bright orange-red color of tea liquor as well as enhance TFs and TRs accumulations in Yunnan Congou black tea YCBT. In contrast, high temperature assisted with TR formation and gave the liquor a glassy appearance. The 20 °C and 25 °C low fermentation temperatures well-maintained polyphenol oxidase activity, promoting TRSI (a TRs fraction) formation, TFs, and TSs, leading to improved LB and tea liquor color. A temperature rise (30 °C, 35 °C, and 40 °C) led to increased catechin oxidative depletion, peroxidase activity, and higher production of TRSII and TBs [151]. Thus, fermentation temperature must be monitored for enzymatic activity [152].

### 6.4. Oxygen and Relative Humidity

Monitoring relative humidity RH and oxygen is crucial to making good-quality black tea. Enzymatic reactions require enough oxygen. Low oxygen levels cause leaf heat and hinder chemical oxidation, which leads to a dull liquor [107]. Low-oxygen and high-temperature fermentation reduces TF content and increases TR content [107,145]. Non-fermented tea contains less volatile flavor components than fermented tea. The essential oil from fermented leaves contains linalool oxides, but fresh leaf homogenates do not. Rapid polyphenol oxidation appears to prevent the production of volatile flavor compounds in tea leaves [72], assuming that 28 °C is ideal [116]. Maintaining high relative humidity (95–98% is vital during fermentation [12,107]. The ruptured leaves must be humidified to stay fresh and cool during the fermentation process in the afternoon when relative humidity is low, and temperature is high. Avoid dry air passage over the leaves, as this interferes with the oxidation rate and causes blackening [107]. Adjusting the fermenting tea pH from 5.5 to 4.5–4.8 decreased thearubigin levels and increased TF levels, which is possibly due to the lower turnover of produced TFs to thearubigins [156]. Excess dhool moisture could hamper aeration and temperature regulation, causing uneven fermentation. Over-withering can make the catechins more concentrated, stopping polyphenol oxidase (PPO) activity and minimizing cell content expression on a macerated leaf surface [157]. Thus, leaf particles may ferment to a significant proportion under oxygen-limiting conditions. It does not appear that moisture loss during withering affects the optimal fermentation period [110,158].

The highest sensory assessment scores, brightness, briskness, and astringency levels were achieved at different fermentation temperatures and durations. Sensory evaluation scores were highest at low fermentation temperature. Thus, maintaining low fermentation temperature for longer fermentation duration produces higher-quality black teas.

## 7. Drying/Firing Stage

Drying stops fermentation by inhibiting enzyme activity, producing dried black tea [41]. Tea particle drying stops oxidation and enzymatic activity, decreases moisture to 3–4% (wb), facilitates handling and transportation, and enhances shelf life [159]. After thermochemical reactions at high temperatures, drying causes dehydration in tea to decrease its moisture level and enhance the taste and aroma of tea. Therefore, measuring tea’s moisture content is crucial to making high-quality tea since it affects both physical and chemical reactions in tea processing and determines its shelf life [60]. During the drying process, the fermented tea color turns from coppery brown to blackish brown [47]. Regulating drying temperature, tea moisture content, and evaporation is crucial for effective drying. Case hardening occurs when tea is dried rapidly enough at high temperatures. The surface of the tea particle dries faster than the core, retaining some moisture and affecting storage quality. However, if a too-low temperature is used for drying the fermented tea, the fermentation will continue. Stewing causes the tea not to dry correctly, affecting its liquoring qualities [47]. It has been reported that heat, but not enzymes, caused the chemical changes during drying. Many of these chemical changes are suitable for the quality of tea, while others are undesirable. When the enzymes (PPO and PO) are deactivated, almost all of the biochemical processes stop. At the initial stage of drying as the drying temperature rises, the enzymes get active, and the reaction proceeds faster, but as the drying temperature rises to the point where enzymes cease to function, the reactions stop. TR formation is likely to persist if the drying temperature is gradually increased. Drying at high temperatures degrades chlorophyll to pheophytin, making tea black. Polyphenols interacting with proteins generate complex compounds at higher temperatures, reducing astringency. Carbohydrates and amino acids react at high temperatures to generate flavor compounds. After a certain level of drying (dry tea), more exposure to heat will degrade quality and cause a burnt taste [160]. Therefore, drying is a crucial phase in the manufacturing process of tea [47].

The two most common dryers used in tea factories are fluidized bed dryers (FBDs) and endless chain pressure (ECP) dryers. The process of FBD involves exposing tea leaves to inlet air that is approximately 140 °C hot. ECP usually involves drying air temperatures of about 100 °C [161]. Hot air drying may decrease volatile flavor elements and quality [162], so researchers are investigating new drying methods. Drying tea leaves at low temperatures preserves their volatile flavor compounds. Tea leaves can be dried at low temperatures using radio frequency, microwave, freeze, and vacuum [161,163,164].

### Factors Influencing the Drying Process

Tea drying and its qualitative attributes are regulated by drying process parameters such as spread thickness, temperature, air flow rate, and drying period [64]. Black tea dried at 110 °C temperature and 1.5 rpm is of excellent quality (Figure 5b). Each lot undergoes a second drying process at 80 °C of low temperature to eliminate 95–97% of the moisture, leading to products that have excellent storage and keeping quality. Since fermentation continues after drying, black tea with more than 6% moisture loses quality, as high-moisture processed tea has a short shelf life [64]. These findings are consistent with [61]. As duration and temperature increase, the biochemical composition and quality of black tea decrease. The most effective combination was found to be 100 °C for 25 min [162].

An increase in amino acid concentrations, loss of volatile substances, binding of tea polyphenols to other tea constituents, carboxylic acid elevation, and Maillard reactions are among the additional changes during the drying process in addition to the removal of moisture. Black tea needs high-temperature drying to develop flavor, color, and aroma. Too-wet dhool can clump and make drying difficult, especially in fluidized bed dryers [165,166]. Compared to the 96 °C dryer temperature, a high inlet drying temperature did not affect the quality of the tea. A higher inlet temperature could enhance the appearance when there are only 40% excellent leaves [167]. Quality may be improved by exposing tea constituents to temperatures of up to 120 °C for less than a minute. With a drying time of under 15 min, stewing was not observed [160]. Microwave drying preserved Vitex negundo Linn’s ascorbic acid content, anthocyanin, equivalent antioxidant activity, and total phenolic content better than other drying methods [168]. Drying Indian gooseberry tea may degrade ascorbic acid content. It was advised to keep hot water low, during its production [169]. The total amount of polyphenols and the color did not change much when an industrial microwave vacuum dryer was used. Thai green tea should be dried at 3600 W for 30 min for excellent physicochemical qualities [170]. Yihong Congou black tea YCBT hot-air-dried exhibits higher sensory scores (Table 4) and better chemical properties than hot roller-dried tea. The hot-air-dried tea enhanced volatile components associated with sweet-flowery flavor, whereas the hot roller-dried tea increased fruity-flavor compounds. In addition, hot air drying decreased most soluble sugar and boosted most free amino acids as compared to hot roller drying [27].

The chemical and sensory properties of black tea were significantly affected by drying methods. Compared to conventional hot air drying, the black tea’s quality was significantly improved, especially the taste and volatile compounds, by the microwave drying, far-infrared drying, halogen lamp drying, and halogen lamp–microwave drying, with the excellent effect from the halogen lamp–microwave, microwave treatments [171] (Table 4).

The effects of a superheated steam dryer (SHS) with drying temperature (120–200 °C) on black tea leaves have been evaluated [172]. It has been noticed that higher drying temperatures enhanced the drying rate and shortened drying periods. Compared to commercial dryer leaves, SHS-dried samples exhibited enhanced color and TPC retention (91.4%) [172]. The sensory quality of the orange, black tea, which was hot air dried, was superior to that of the traditional outdoor-sunlight-dried tea, with a harmonized sweet–mellow flavor and fruity aroma. After hot air drying, in tea leaves and peel the polyphenols content and other quality components were much greater than sunlight drying. Antioxidant capacity was higher in hot-air-dried tea than in sunlight-dried tea. Analyzing the effects (40 °C, 45 °C, 50 °C, and 60 °C) of drying temperatures on orange black tea quality revealed a gradual decrease in quality with drying temperature, with the most noticeable decrease at 60 °C. In orange peel and black tea, 40 °C-dried tea showed the best aroma coordination, sweet–mellow taste, fruit flavor, and functional, active substance retention. When the fermented tea leaves are exposed to temperatures that are too low or too high, the tea particles lead to stewing and hardening respectively, which decreases tea liquor quality. In general, tea leaves exposed to temperatures ranging from 90 °C to 140 °C are determined to be adequate [173]. Drying temperature and duration play a crucial role and must be carefully monitored.

## 8. Storage Method and Duration

Chemical reactions in storage swiftly remove harshness and greenness from the final tea product. Tea stays flavorful and healthy for over a year in cool conditions and away from air and moisture [174]. Broadly, black tea quality is the sum of all desired qualities that determine its market value. The quality of black tea is determined by briskness, flavor, aroma, color, and strength, as well as a chemical constituent concentration in the brew that affects tea quality. Catechins in black tea oxidize to TFs and TRs during processing. Tea tastes bitter because of the 1, 3, 7-methylxanthine in it, so it has been concluded that long-term improper storage of black tea significantly reduced its quality [175]. The storage impact on the taste quality and chemical profile of Keemun black tea after 1, 2, 3, 4, 5, 10, 17, and 20 years of storage was evaluated [176]. During 10-year storage, the significant polyphenols declined, although theobromine and caffeine remained stable. The astringency, umami, and bitterness intensities were inversely correlated with years of storage, but the sweetness was positively correlated. A positive correlation between fatty acid content to sweetness and storage time was found. In Northeast India, the black tea color profile changed significantly during storage. Following one month of production, some pigments were enhanced and sustained for 8 months without considerable change [177]. Tea may lose flavor and astringency during several months of transportation and storage, leading to unpleasant attributes. Oxygen is consumed by black tea during this period, suggesting that oxidative deterioration may grant changes in aroma quality after processing [178]. Compared to tea stored under normal conditions, the TF level of accelerated-storage black tea slightly dropped. The accelerated-storage black tea showed a slight rise of TRs of 13.71% in the first month and a slight reduction of TRs of 11.81% in the second month. Total color increased in accelerated storage samples (4.73) in the first month and decreased (4.17) in the second month [179]. It has been observed that black tea that was stored improperly for an extended period lost its qualitative properties [175]. However, prolonged storage, particularly in conditions where moisture and light exist, degrades the quality of the tea, which eventually results in “softness”, or lacking briskness and having a “flat” taste [180]. Black tea deteriorates by losing astringency and flavor and sometimes finding undesirable “taints” due to autoxidative reactions and lipid hydrolysis that reduce sugars, TF, photosynthetic pigments, amino acids, and some volatile aliphatic elements and raise volatile phenolic and non-dialyzable pigments. Heat and moisture accelerate these reactions. Even though lipid oxidation is negligible except under dry and hot conditions, it is hypothesized that oxidation of free fatty acids released during storage occurs during brewing and has a significant impact on tea liquor quality [181].

A study [182] reported that under all conditions, undesirable changes occurred, but temperature and moisture content accelerated deterioration. According to the tea taster’s sensory criteria, high moisture content is more detrimental to high temperature. A 15-day storage period did not affect black tea’s antioxidant capacity and total phenolic content. Black tea’s total phenolic content and DPPH scavenging activity were negligibly varied at storage temperatures of 4 °C, 9 °C, and 25 °C [183]. The black tea’s DPPH scavenging activity decreased after three months and its total phenolic content remained stable at 25 °C storage even after six months [184]. Kombucha made with sugared black tea can be kept for up to four months at refrigeration temperature. The kombucha’s antioxidant qualities will be lost after this time [185].

Tea samples stored at accelerated conditions had lower brightness than control samples. Caffeine levels decreased in tea stored in accelerated storage conditions compared to ordinary storage, whereas moisture content increased compared to the control group. Samples stored under accelerated storage had an 8–9 times higher microbial population than samples stored normally.

## 9. Conclusions

This study reviewed the existing evidence on tea processing techniques and their influence on phytochemicals and sensory qualities. Literature indicates that black tea quality relies on its chemical composition, particularly harvested shoot flavanols, and on its handling, processing, and storage. After plucking, numerous biochemical and physiological processes take place during processing. The most crucial process is fermentation in the main quality factor determination of black tea, among all the stages of manufacturing. It was found that the plucking technique affected the sensory attributes and chemical quality characteristics of black teas. There was a significant difference between machine-picked and hand-picked teas regarding caffeine, brightness, TF, flavor index, and Group II volatile flavor compounds (VFCs). A bud and two leaves are highly suggested since fine plucking produced significant TF, caffeine, and total water-soluble solids. Only a bud and two leaves had significant amounts of TF, and they also had optimal levels of polyphenol oxidase activity and catechin concentration. In terms of caffeine, TF, brightness, aroma, and sensory ratings, black teas from short plucking intervals were preferable to those plucked from long rounds. Wet, warm seasons are characterized by low-quality black tea with high production and quick growth, whereas dry and cool seasons lead to high black tea quality with a slower shoot growth rate. Black tea processed from spring to summer has considerably more caffeine and soluble sugar. Although there is no set withering duration, 14 to 18 h at room temperature is generally regarded as optimal. Significant TR content, tea liquor color, and sensory score were found in leaves that were withered for 16 h. The color properties of tea liquor and their corresponding pigments TFs, TRs, and theabrownins were shown to rise dramatically with increased rolling time. The minimum bitter intensities and maximum umami were found in black tea rolled for 90–100 min. As fermentation progresses, TF and TR concentrations rise, and other desired quality attributes peak and decline as the process is prolonged. The optimal fermentation conditions were found to be 20–30 °C, 85–90% RH, and 6–8 h. Drying black tea at 110 °C for 10 min, followed by 85 °C for 3.5 h at 1.5 rpm dryer speed, results in high-quality tea. During this, it is also vital to sustain the tea’s moisture content; otherwise, it will quickly deteriorate. Tea loses its quality over time if stored for too long, especially if exposed to light and moisture. Over time, the tea becomes “soft,” meaning that it lacks briskness and has a “flat” taste. The final product’s quality is a key consideration in the tea production and handling process. The black tea’s handling (plucking method, standard, season, and interval) should be given due attention, and the processing also requires precise control of time, humidity, temperature, and air flow rate. Evidently, developing an advanced mechanism to regulate such factors under different circumstances would be necessary to maintain the quality. Consequently, the careful consideration of processing steps and storage systems, including plucking, is fundamental to achieving high-quality black tea. Employing advanced mechanisms is key to optimizing these factors and ensuring the desired characteristics in the final product. Otherwise, it is hard to achieve the desirable qualities of black tea.

## Figures and Tables

**Table 1:** Effect of different plucking methods on chemical composition of black tea.

Parameters (Chemical Quality)	Hand Plucked	Shear-Plucked A	Shear-Plucked B		Hand Plucked	Shear-Plucked	
Theaflavin (%)	0.78	0.71	0.76	[53]	13.16 μmols g^−l^	12.81 μmols g^−l^	[50]
Thearubigins (%)	7.60	8.10	7.90		13.64	14.12	
High polymerized substances (%)	7.10	7.70	7.30				
Total liquor color	2.60	2.50	2.60		3.16	3.23	
Water extract (%)	41.90	40.70	41.60				
Crude fiber (%)	15.40	14.90	15.20				
Caffeine (%)	3.30	3.00	3.10		2.72	2.57	
Lipid (%)	3.00	3.30	3.10				
Protein (%)	16.0	16.6	16.2				
Taster’s score							
A	33.00	30.00	32.00				
B	36.00	32.00	33.00				
Biochemical constituents							
Total catechin (%)	18.4	17.8	18.2				
Total polyphenols (%)	27.7	26.6	27.5				
PPO activity (U/mg protein)	24.4	23.1	23.0				
Total lipid (%)	6.3	7.7	7.3				
Total protein (%)	14.1	15.3	15.1				
Fatty acids							
Palmitic (16:0)	15.3	14.6	14.9				
Stearic (18:0)	8.0	7.7	8.0				
Oleic (18:1)	7.4	8.8	8.1				
Linoleic (18:2)	20.7	22.5	21.3				
Linolenic (183)	37.2	37.0	36.8				

**Table 2:** Variation in chemical composition of black tea due to plucking standard, interval, and rolling time.

Parameters	Plucking Standard							Plucking Interval					Rolling Time				
	Bud	1 Leaf +Bud	2 Leaves +Bud	3 Leave +Bud	4 Leaves +Bud	5 Leaves +Bud		5 Days	7 Days	9 Days	11 Days		50/Min Rolling	75/Min Rolling	100/Min Rolling	125/Min Rolling	
Caffeine %	3.89	3.42	2.11	1.56	1.29	1.22	[66]	4.4	4.79	4.3	3.81	[67]					[68]
Theaflavins µM/g or %	23.21	33.43	34.71	29.99	27.33	22.42		1.12%	1.22%	1.2%	1.4%		0.3007 ± 0.0046%	0.3698 ± 0.0047%	0.3968 ± 0.0013%	0.3825 ± 0.0098%	
Thearubigins %	8.26	12.93	17.81	18.19	18.99	16.68		13.5	13.9	14.9	15.74		3.0475 ± 0.0529	3.5088 ± 0.1417	4.3983 ± 0.0949	5.1962 ± 0.0681	
Total water-soluble solids %	48.2	50.6	47.9	45.00	41.9	42.6		44.3	44.4	42.5	41.44						
Ash %	7.21	6.78	6.1	6.38	6.37	6.65		5.95	6.15	6.16	6.15						
Crude fiber %	6.76	8.12	10.22	13.68	14.86	16.65		6.7	7.00	9.4	10.15						
Sum of Group I	3.61	4.47	4.63	4.86	5.23	5.74											
Sum of Group II	8.18	8.30	6.94	5.50	5.06	4.54											
FI. (II/I)	2.26	1.86	1.50	1.13	0.97	0.79											
TF/TR %								0.08	0.09	0.09	0.09						
Taster’s Evaluation								Very Good	Good	Good	Fair						
Theabrownin%													5.5444 ± 0.0535	6.2834 ± 0.0373	6.4246 ± 0.0509	6.707 ± 0.0509	

**Table 3:** Effect of different fermentation time on quality of black tea.

Time (min)	TFs ^1^ (%)	TR ^2^ (%)	Caffeine (%)	C ^3^ (%)	EC ^4^ (%)	EGCG ^5^ (%)	ECG ^6^ (%)		Time (min)	Color	Taste	Aroma	Strength	Infusion	
15	1.61	10.5	2.71	0.632	0.310	8.15	0.574	[13]	135	Dull	Fair	Fair	Weak	Dark/Dull	[64]
30	2.29	12.5	2.93	0.207	0.923	4.11	0.377		140	Dull and light red	Fair	Good	Weak	Mixed	
45	2.59	12.7	2.90	0.240	0.914	3.23	0.289		145	Dull and light red	Fair	Good	Strong	Even and Bright	
60	2.43	13.1	2.74	0.271	0.844	2.83	0.231		165	Dull	Fair	Very Good	Strong	Coppery	
75	2.38	13.3	2.88	0.250	0.787	1.77	0.165		175	Light Red	Good	Excellent	Very Strong	Dark or Dull	
90	2.29	14.2	2.86	0.321	0.417	2.03	0.144		305	Bright Red	Very Excellent	Excellent	Very Strong	Dark and Dull	
105	2.28	13.1	2.68	0.304	0.750	1.21	0.098								
120	2.20	12.9	2.81	0.295	0.614	1.46	0.084								
135	2.03	13.2	2.86	0.341	0.870	1.63	0.084								
150	1.98	12.8	2.51	0.249	0.636	1.42	0.055								
165	2.01	12.8	2.76	0.252	0.434	1.13	0.054								
180	1.96	13.3	2.72	0.23	0.867	1.25	0.052								

^1^ TFS: theaflavins; ^2^ TR: thearubigins; ^3^ C: catechins; ^4^ EC: epicatechins: ^5^ EGCG: epigallocatechin gallate; ^6^ ECG: epicatechin gallate.

**Table 4:** Effect of different drying methods on black tea quality.

Different Drying Methods									
Parameters	HDT ^1^	FDT ^2^	MDT ^3^	LDT ^4^	MLDT ^5^		HA ^6^	HR ^7^	
Dry tea color (10%)	84.0 ± 0.5	89.0 ± 1.7	88.3 ± 1.2	88.8 ± 1.6	88.8 ± 2.4	[171]	90 ± 0.5	88 ± 0.7	[27]
Dry tea streak (10%)	83.3 ± 0.6	83.5 ± 0.5	82.3 ± 2.1	82.7 ± 1.5	83.0 ± 2.0		86 ± 1.0	90 ± 0.8	
Liquor color (10%)	89.5 ± 1.3	89.3 ± 3.1	87.7 ± 1.5	88.2 ± 1.3	90.0 ± 1.5		91 ± 0.5	89 ± 0.6	
Aroma (30%)	81.5 ± 0.5	84.0 ± 1.8	90.0 ± 2.2	86.2 ± 2.0	91.3 ± 1.8		89 ± 0.6	87 ± 0.4	
Taste (30%)	81.8 ± 1.0	84.2 ± 2.1	90.0 ± 1.8	87.0 ± 2.0	91.5 ± 1.5		89 ± 0.5	85 ± 0.7	
Infused leaf (10%)	84.0 ± 0.5	84.2 ± 1.0	83.0 ± 1.0	83.7 ± 1.0	83.8 ± 2.0		89 ± 0.8	88 ± 0.5	

^1^ HDT: hot-air-dried black tea; ^2^ FDT: far-infrared-dried black tea; ^3^ MDT: microwave-dried black tea; ^4^ LDT: halogen-lamp-dried black tea; ^5^ MLDT: halogen lamp–microwave-dried black tea; ^6^ HA: hot-air-dried black tea; ^7^ HR: hot-roller-dried black tea.

## Data Availability

Data are contained within the article.
